# Therapeutic Poetry Program for Indigenous People Living with Dementia

**DOI:** 10.1002/alz.094748

**Published:** 2025-01-09

**Authors:** Antonio Paniagua Guzman

**Affiliations:** ^1^ University of Minnesota, Duluth, MN USA

## Abstract

**Background:**

Due to its accessibility and connection with cognitive and linguistic abilities, poetry has been used to study diverse characteristics of experiential and emotional outcomes of people living with dementia (PLWD). Previous studies have shown that the practice of poetry has the potential of improving health, cognitive, and communicational outcomes of PLWD. Also, it is supportive in understanding the experiences and needs of PLWD and has proved to be an effective mechanism to improve the relationships between PLWD and their caregivers. Even though much needed poetry interventions have been developed and implemented to serve diverse populations in the United States, no culturally safe programs specifically for Indigenous PLWD have been developed.

**Method:**

Using a community‐based participatory research (CBPR) framework and Indigenous methodologies, this study is based on the development and implementation of a pilot therapeutic poetry program for Indigenous PLWD and their caregivers in an Indigenous Nation in Minnesota, through the adaptation of existing techniques used to deliver poetry‐based therapy for PLWD. Aligned with CBPR principles, this pilot program will be facilitated and evaluated by community members.

**Results:**

N/A

**Conclusion:**

With the participation of 12 Indigenous dyads, this project aims to (a) implement a sustainable program that can be replicated in other communities; (b) assess the effects of the program on dyads’ relationships, quality of life, intra‐community relationships, cultural performance, and Indigenous identity; and (c) identify mechanisms that improve quality of life, quality of care, relationships, and the regulation of behavioral issues for PLWD in the Indigenous context.

